# PNPase is involved in the coordination of mRNA degradation and expression in stationary phase cells of *Escherichia coli*

**DOI:** 10.1186/s12864-018-5259-8

**Published:** 2018-11-29

**Authors:** Clémentine Dressaire, Vânia Pobre, Sandrine Laguerre, Laurence Girbal, Cecilia Maria Arraiano, Muriel Cocaign-Bousquet

**Affiliations:** 1 Instituto de Tecnologia Química e Biológica António Xavier, Universidade Nova de Lisboa, Av. da República, 2780-157 Oeiras, Portugal; 2 LISBP, Université de Toulouse, CNRS, INRA, INSA, Toulouse, France

**Keywords:** RNA decay, PNPase, RNase R, *E. coli*, Transcriptome, Gene expression regulation

## Abstract

**Background:**

Exoribonucleases are crucial for RNA degradation in *Escherichia coli* but the roles of RNase R and PNPase and their potential overlap in stationary phase are not well characterized. Here, we used a genome-wide approach to determine how RNase R and PNPase affect the mRNA half-lives in the stationary phase. The genome-wide mRNA half-lives were determined by a dynamic analysis of transcriptomes after transcription arrest. We have combined the analysis of mRNA half-lives with the steady-state concentrations (transcriptome) to provide an integrated overview of the in vivo activity of these exoribonucleases at the genome-scale.

**Results:**

The values of mRNA half-lives demonstrated that the mRNAs are very stable in the stationary phase and that the deletion of RNase R or PNPase caused only a limited mRNA stabilization. Intriguingly the absence of PNPase provoked also the destabilization of many mRNAs. These changes in mRNA half-lives in the PNPase deletion strain were associated with a massive reorganization of mRNA levels and also variation in several ncRNA concentrations. Finally, the in vivo activity of the degradation machinery was found frequently saturated by mRNAs in the PNPase mutant unlike in the RNase R mutant, suggesting that the degradation activity is limited by the deletion of PNPase but not by the deletion of RNase R.

**Conclusions:**

This work had identified PNPase as a central player associated with mRNA degradation in stationary phase.

**Electronic supplementary material:**

The online version of this article (10.1186/s12864-018-5259-8) contains supplementary material, which is available to authorized users.

## Background

Intracellular RNA levels are a result of both transcription and degradation rates. Although transcription is important, RNA degradation is also a key factor in the regulation of gene expression [1–3]. Ribonucleases can either degrade the RNA internally (endoribonucleases) or degrade the RNA from one of the extremities (exoribonucleases). In *Escherichia coli* RNA degradation involves mainly two endoribonucleases (RNase III and RNase E) and three 3′-exoribonucleases (PNPase, RNase II and RNase R) [1]. In this bacterium, no 5′-exonuclease activity has been detected unlike in *Bacillus subtilis* [4, 5]. These RNases can either act alone or they can form RNA degradation complexes with other proteins [1, 3]. In prokaryotes there are two main RNA degradation pathways. One starts with an endoribonucleolytic cut followed by the exoribonucleolytic degradation of the smaller fragments and the other only requires exoribonucleases for the degradation of the full-length RNA [1, 3, 6]. Therefore, exoribonucleases are crucial for RNA degradation.

PNPase is a phosphorolytic exoribonuclease but under some conditions such as low inorganic phosphate or in the absence of poly(A) polymerase, PNPase can add polynucleotide tails to RNAs [7–9]. PNPase activity is blocked by double stranded RNA structures [10], but it can form complexes with other proteins allowing it to degrade through extensive structured RNA [1]. RNase II and RNase R are both hydrolytic exoribonucleases and belong to the RNase II family of enzymes [11]. RNase II degrades only single stranded RNA while RNase R is able to degrade structured RNA as long as there is a 3’end overhang. RNase R is a stress-induced protein [12–14] and it is the only exoribonuclease able to degrade highly structured RNA without the help of other factors [14]. RNase II, RNase R and PNPase seem to have some overlapping roles in the cell. The deletion of any of the exoribonucleases does not affect cell viability and a double mutant RNase R/RNase II is also viable. However, the double mutants PNPase/RNase R and PNPase/RNase II are not viable [15, 16].

All these exoribonucleases have been extensively studied, mostly in exponential phase of growth in laboratory conditions. However, both RNase R and PNPase are active in other conditions. As an example, PNPase and RNase R are involved in the virulence process in several organisms [17–21]. More particularly, PNPase and RNase R are active in *E. coli* in stationary phase. Under such conditions, PNPase participates in the degradation of small RNAs [22–24]. The levels of RNase R increase in stationary phase [12]. In exponential phase most of the RNase R is associated with ribosomes [25, 26] and therefore there is less amounts of enzyme available for RNA degradation. On the other hand in stationary phase RNase R is no longer associated with the ribosomes and the protein level is also increased due to protein stabilization [27]; therefore in stationary phase there is more RNase R available for RNA degradation.

In bacteria the role of exoribonucleases in RNA degradation has been mostly studied at the mechanistic level, with only a few genome-wide analyses of the exoribonuclease activity [28–33]. To date, in *E. coli*, only four genomics studies of exoribonuclease activity were reported and they were all done in exponential phase [28, 29, 32, 33]. These large-scale studies generally consider transcriptomic analysis which represents the steady-state levels of the mRNAs in the different RNase mutants. Transcriptomic data do not necessarily represent only the direct result of the processing and degradation activities of RNases since there are many possible indirect effects (e. g. changes in transcription). Although RNase activity is expected to directly modify RNA decay, genome wide-quantification of RNA stability is only rarely undertaken.

In this work we studied the role and the overlap of RNase R and PNPase exoribonucleases in stationary phase. We compared in this phase the mRNA half-lives for *E. coli* MG1655 and the mutants deleted for PNPase or RNase R. We then combined the analysis of mRNA stabilities with the steady-state concentrations (transcriptome) to provide an integrated overview of the in vivo activity of these exoribonucleases at the genome-scale.

## Results

### mRNAs are very stable in stationary phase

**Fig. 1:**
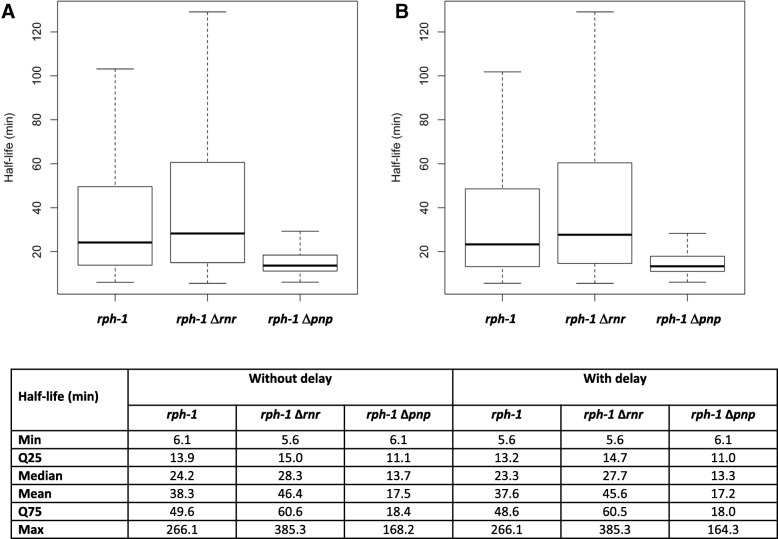
Statistics associated with the calculated half-lives of 2856 genes in the *rph-1* control strain and the two RNase mutants. Two different models were tested: the model without delay corresponding to classical linear model fitted on log concentration of mRNA **a** and the model with delay previously described [35] corresponding to a piecewise model composed of a stable baseline level during the delay followed by an exponential decay **b**. Results for each strain are represented as boxplots and the related statistics are described below in numbers

 The deletion strains of RNase R or PNPase (the *rph-1* Δ*rnr* and *rph-1* Δ*pnp* double mutant strains, respectively) were constructed in the *E. coli* K-12 MG1655 background referred as the *rph-1* control strain. The *E. coli rph-1* control strain and the *rph-1* Δ*rnr* double mutant displayed similar growth profiles in LB medium while the *rph-1* Δ*pnp* double mutant led to slower growth. Since this work aimed at comparing the role of PNPase and RNase R exoribonucleases during the stationary phase of growth, samplings for genome scale mRNA stabilities were performed at late stationary phase in standardized conditions, i.e. 3 h after the growth stopped (Additional file 1: Figure S1). Using our calculation and quality control method (see material and methods for details), 2856 half-lives were available for all the three strains. This dataset was retained for further analysis (Fig. 1a). Half-lives have been determined with classical linear model fitted on log concentration of mRNA (see example of a set of 50 randomly selected mRNAs in Additional file 2: Figure S2). The half-lives obtained (Fig. 1, median value > 13 min) were higher than previously published in the *rph-1* control strain and the *rph-1* Δ*pnp* double mutant [2, 29, 34], however our growth conditions were different (prolonged stationary phase versus exponential phase), and this could justify in part the discrepancy in the results. We also hypothesized that a delay in the transcription arrest during the mRNA half-life measurement could lead to an increase in the determination of the general values of half-lives. Such a delay, probably due to RNA polymerase elongation activity in presence of rifampicin, was first demonstrated by Chen and coworkers [34] and modelled more recently [35]. We have subsequently re-estimated the half-lives taking into account the delay using a model composed of a stable baseline level during the delay followed by an exponential decay (as described by [35]). The boxplot obtained with the delay (Fig. 1b) is similar to the one obtained without delay, meaning that the half-life values are very similar with and without delay (see correlation coefficient close to 1 in Additional file 3: Figure S3).

The values of half-life were confirmed by Northern-blot experiments with 3 different mRNAs (*osmB*, *ompA* and *nlpl*) measured in the 3 strains. Although the absolute values differed, the two sets of mRNA half-lives were in the same range of magnitude (Additional file 4: Figure S4). A perfect match was even found for the half-life of *osmB* in the 3 strains.

The high mRNA half-lives obtained for the control strain and for the two exoribonuclease mutant strains were not related neither to the measurement method nor to the fitting estimation. These values were thus rather linked to the *E. coli* physiology in prolonged stationary phase. Here we demonstrated that mRNAs are very stable in non-growing cells. Stabilization of mRNAs at zero growth is in agreement with the recent demonstrations of mRNA stabilization at low growth rates [2, 34, 36]. Overall our results show an important stabilization of mRNAs associated to *E. coli* adaption to unfavorable growth conditions.

### Only a few mRNAs are stabilized in the *rph-1* Δ*rnr* and *rph-1* Δ*pnp* double mutants

It was expected that mRNAs would be stabilized at the genome-wide level in the absence of the ribonucleases RNase R or PNPase. The half-lives in the *rph-1* Δ*rnr* and *rph-1* Δ*pnp* double mutants were compared to the *rph-1* control strain at the global level (comparison of statistical distribution of data) and for individual mRNAs applying the statistical test defined in the material and methods section. In the *rph-1* Δ*rnr* double mutant, the median of the mRNA half-lives was 28.3 min while for the *rph-1* control strain the median of the half-lives was 24.2 min (Fig. 1). Between *rph-1* Δ*rnr* and the *rph-1* control there was no mRNA with a significant variation of stability suggesting only a small systematic increase in global mRNA stability in the *rph-1* Δ*rnr* double mutant*.* In the *rph-1* Δ*pnp* double mutant there is a global destabilization (see the lower mean and median half-lives compared to the *rph-1* control in Fig. 1) but 214 mRNAs were found to be significantly stabilized. These stabilized mRNAs belonged to several functional categories. Functional enrichment analyses processed with AMIGO or KEGG Mapper revealed a significant enrichment of mRNAs involved in the central carbon metabolism. The functional categories “glycolysis” and “TCA cycle” were indeed enriched. More specifically, important genes of these processes were stabilized in the *rph-1* Δ*pnp* double mutant (*pgi, pfkA, pgk, pck, acp, gltA, sdaA, sucA, sucC, ldhA, agp, talB* and *yccx*). Interestingly, Bernstein et al. similarly reported the stabilization of *pgi, pfkA, pgk, pckA, gltA* and *talB* in a *rph-1* Δ*pnp* double mutant strain compared to a *rph-1* strain in exponential phase [29] (data for the other genes mentioned here were not available). Recent papers also pointed out the link between PNPase and the regulation of the expression of genes related to the central carbon metabolism [33, 37].

We did not observe any massive stabilization in the *rph-1* Δ*rnr* and *rph-1* Δ*pnp* double mutants, neither taking in consideration the results of mRNA stability all together nor at the individual scale.

### mRNAs are extensively destabilized in the *rph-1* Δ*pnp* double mutant

Many mRNAs displayed decreased half-lives in the *rph-1* Δ*pnp* double mutant compared to the *rph-1* control strain. In comparison only five mRNAs, namely *osmB*, *cstA*, *uspB*, *ycgB* and *ychH*, were destabilized in the *rph-1* Δ*rnr* double mutant*.* Deletion of *pnp* thus led to an overall loss of stability, suggesting that the PNPase could be involved in a protective mechanism of mRNAs.

Modifications of mRNA stabilities in the *pnp* mutant may be related to an indirect effect notably via sRNAs. PNPase was shown to have a significant role in the degradation and also in the protection of sRNAs [23, 24, 28]. Considering that a single sRNA can regulate the expression of several targets [24], it is possible that, in the absence of PNPase, the stability of sRNAs would be changed leading to the destabilization of their targets. We obtained the levels of ncRNAs in stationary phase by RNA-Seq and found important variations in ncRNA levels in the *rph-1* Δ*pnp* double mutant in comparison to the *rph-1* control strain (Table 1). Indeed many ncRNA expressions were up- or down-regulated in the *rph-1* Δ*pnp* double mutant. This is notably the case for the sRNA CsrB with a 7 fold higher level in the *rph-1* Δ*pnp* double mutant*.* PNPase was previously demonstrated to be involved in the degradation of the sRNA CsrB in *Salmonella* [38] but not yet in *E. coli*. By sequestrating CsrA [39], CsrB is expected to control various CsrA-targeted mRNAs. The *flhDC* mRNA protected by CsrA from RNase E-mediated cleavage [40] as well as 696 mRNAs previously shown as destabilized by CsrA attenuation [41] were indeed destabilized in the *rph-1* Δ*pnp* double mutant*.* A large number of the mRNAs destabilized in the *rph-1* Δ*pnp* double mutant (44%) could thus be related to a CsrA-dependent destabilization.

**Table 1:** List of the ncRNAs differentially expressed in the *rph-1* Δ*pnp* double mutant vs the *rph-1* control strain in stationary phase. ncRNAs were quantified with the RNAseq technology in the *rph-1* Δ*pnp* double mutant and the *rph-1* control strain. FC is the fold-change of expression in the *rph-1* Δ*pnp* double mutant compared to the *rph-1* control

ncRNA	Description	FC *rph-1* Δ*pnp* double mutant vs *rph-1* control
agrA	Inactive antisense sRNA	6.1
arcZ	sRNA positive antisense regulator of rpoS; binds Hfq	0.5
arrS	Antisense sRNA regulator of gadE and acid resistance; GadE-regulated	0.4
csrB	CsrA-binding sRNA. antagonizing CsrA regulation; blocks the CsrA binding of hundreds of mRNAs	7.0
cyaR	sRNA effector of ompX mRNA instability, cAMP-induced; hfq-dependent	2.3
dsrA	Regulatory sRNA enhances translation of rpoS; component of acid resistance regulatory circuit; also antagonist of H-NS function by decreasing H-NS levels	0.5
gadY	sRNA regulator of gadAB transcriptional activator GadX mRNA	3.2
gcvB	GcvB sRNA gene divergent from gcvA; represses oppA, dppA, gltI and livJ expression; regulated by gcvA and gcvR; this is gcvB-L, terminated at T2, 90% of gcvB RNA is from gcvB-S, encoding a 134 nt RNA terminating at T1	5.3
glmY	sRNA activator of glmS mRNA, glmZ processing antagonist	0.5
glmZ	sRNA antisense activator of glmS mRNA, Hfq-dependent	6.1
micA	sRNA regulator of ompA, lamB, ompX and phoP, Hfq-dependent	1.7
micF	Regulatory antisense sRNA affecting ompF expression; member of soxRS regulon	1.7
omrA	sRNA downregulating OM proteins and curli; positively regulated by OmpR/EnvZ; binds Hfq	0.3
omrB	sRNA downregulating OM proteins and curli; positively regulated by OmpR/EnvZ; binds Hfq	0.2
psrD	Novel sRNA, function unknown	3.7
rdlA	Antisense sRNA RdlA affects LdrA translation; proposed addiction module in LDR-A repeat, with toxic peptide LdrA	0.5
rnpB	RNase P, M1 RNA enzyme component; involved in transfer RNA and 4.5S RNA-processing	5.7
rprA	Positive regulatory antisense sRNA for rpoS translation	0.6
rybB	sRNA effector of ompC and ompW mRNA instability; requires Hfq	0.4
rydC	sRNA regulator of csgD and yejABEF	2.3
ryeA	Novel sRNA, function unknown	1.9
ryjB	Novel sRNA, function unknown	2.1
sgrS	sRNA that destabilzes ptsG mRNA; regulated by sgrR	9.8
sibA	Antisense sRNA regulator of toxic IbsA protein; in SIBa repeat	26.0
sibB	Antisense sRNA regulator of toxic IbsB protein; in SIBb repeat	3.2
sibC	Antisense sRNA regulator of toxic IbsC protein; in SIBc repeat	11.3
sibD	Antisense sRNA regulator of toxic IbsD protein; in SIBd repeat	13.0
sibE	Antisense sRNA regulator of toxic IbsE protein; in SIBe repeat	1.9
sokB	Antisense sRNA blocking mokB, and hence hokB, translation	0.5
sokX	Antisense sRNA, function unknown	3.2
ssrA	tmRNA, 10Sa RNA; acts as tRNA-Ala and mRNA template for tagging proteins resulting from premature transcription termination for degradation, a process known as trans-translation	1.4
symR	sRNA destabilizing divergent and overlapping symE mRNA	0.5

### Massive reorganization of genome-wide mRNA levels in the *rph-1* Δ*pnp* double mutant

**Fig. 2:**
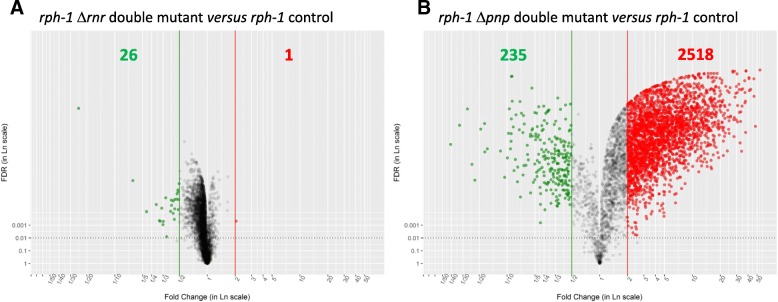
Volcano representation of the transcriptomic comparisons of the RNase mutant strains with the *rph-1* control strain. Volcano plots representing the transcriptome results corresponding to the *rph-1* Δ*rnr* double mutant (left side) and the *rph-1* Δ*pnp* double mutant (right side) compared to the *rph-1* control strain. Genes associated to a FDR lower than 1% (represented by the scattered horizontal line) and a fold change lower than 0.5 or higher than 2 represented the down- (green spots) and up-regulated genes (red spots), respectively

Modification of RNase activity in the different RNase mutants is expected to directly change mRNA stabilities, therefore altering mRNA concentrations. However, many indirect effects on mRNA concentrations are also likely to occur, for instance when RNases targeted the stability of transcriptional regulators [32, 33]. We have notably found in this work that 70 transcriptional regulators (among the 162 identified according to RegulonDB) exhibited a modified stability in the *rph-1* Δ*pnp* double mutant (Additional file 5: Table S1). Therefore, in order to decipher the role of RNase R and PNPase, we have estimated the genome-scale mRNA concentrations by transcriptomic analysis and compared the data in the different strains. Raw data of steady-state transcriptome before addition of rifampicin were recomputed with a RMA-derived method (see material and methods for details). Dramatic changes were observed in the *rph-1* Δ*pnp* double mutant whereas only a minor transcriptional reorganization occurred in the *rph-1* Δ*rnr* double mutant (Fig. 2)*.* Indeed, applying the statistical criteria as defined in material and methods, only 27 genes were differentially expressed between the *rph-1* Δ*rnr* double mutant and the *rph-1* control strain whereas 2753 displayed significantly different levels between the *rph-1* Δ*pnp* double mutant and the *rph-1* control. Most of the genes were up-regulated in the *rph-1* Δ*pnp* double mutant compared to the *rph-1* control strain (Fig. 2). Our transcriptome results are in agreement with recently published RNA-Seq data showing that, in exponential phase, PNPase deletion led to more up-regulated transcript concentrations compared to RNase R deletion [33]. Such changes in mRNA levels in the *rph-1* Δ*pnp* double mutant were corroborated by the 4-fold increase of the total mRNA concentration and by the 2-fold increase of the total RNA (mRNA, rRNA and tRNA) content in this strain in comparison to the *rph-1* control strain (Additional file 6: Table S2).

Overall, the deletion of PNPase led to a massive reorganization of the gene expression most probably due to indirect transcriptional regulations. The global up-regulation of mRNA concentrations observed in the *rph-1* Δ*pnp* double mutant is in the opposite direction to the down-regulation of mRNA stabilities also found in this strain.

### PNPase affects the relationship between mRNA stability and concentration

**Fig. 3:**
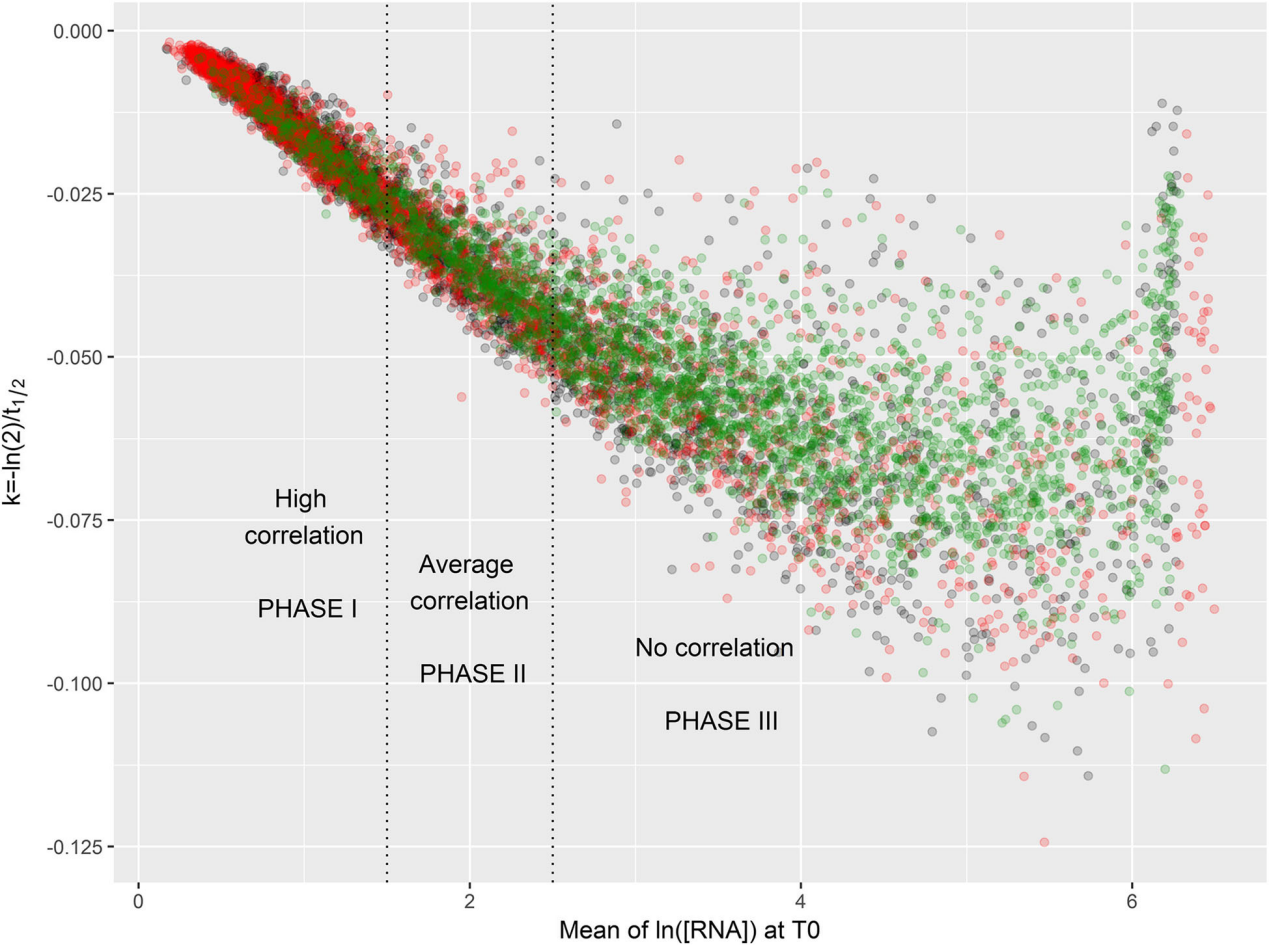
Plot of the degradation rate constant as a function of the mRNA concentration for the *rph-1* control strain, the *rph-1* Δ*rnr* and *rph-1* Δ*pnp* double mutants. For the 2856 genes with available half-lives in the three strains, the degradation rate constant k was plotted against the average mRNA concentration (normalized value) at T0 (before rifampicin addition). The *rph-1* control is represented in black, the *rph-1* Δ*rnr* double mutant in red and the *rph-1* Δ*pnp* double mutant in green. Appling a K-means clustering algorithm, three correlation phases were distinguished: phase I, zone of highly significant (*p*-value < 10^− 100^) and very strong negative correlation (correlation coefficient ~ − 0.8) associated with low mRNA concentrations; phase II at intermediary mRNA concentrations with strong and significant negative correlation (correlation coefficient ~ − 0.65 and *p*-value < 10^− 15^); phase III, lack of correlation at high mRNA concentrations (correlation coefficient ~ 0)

The role of exoribonuclease activities was thus complex, combining effects on both mRNA stability and mRNA concentration. This suggests a delicate balance between regulations of mRNA stability and concentration in cells. Until now, we have compared the behavior of genome-wide mRNA concentrations and half-lives between two strains. We focus now on the relationship between mRNA concentrations and half-lives within the same strain. The overall relationship between mRNA stability and concentration characterizes the in vivo activity of the degradation machinery (at the level of enzyme-substrate interaction) [42]. In order to determine the impact of PNPase or RNase mutations on the in vivo degradation activity, we have analyzed the stability - concentration relationship for all mRNAs of each strain using a genome-wide correlation analysis. We have combined on Fig. 3, the three plots of the degradation rate constant k (k = ln2/ half-life) versus the mRNA concentration obtained for the *rph-1* control strain and the *rph-1* Δ*rnr* and *rph-1* Δ*pnp* double mutants, respectively. This figure allowed the visualization of a global negative relationship between degradation rate constant and the mRNA concentration for each individual strain but the level of correlation differed with the mRNA concentration. Three correlation phases were differentiated using a K-means clustering algorithm on the combined three datasets (see the correlation coefficients and *p*-values in the legend of Fig. 3). In phase I, for the lowest mRNA concentrations, the correlation was very strong; in phase II for intermediary mRNA concentrations, the correlation was less strong and in phase III for the highest mRNA concentrations, the correlation was not significant. Phases determined with combined data of the three strains were also relevant for individual strains (see correlation coefficient for individual strains in the three phases in Table 2). For each of the three strains, most of the genes showed a negative correlation between the degradation rate constant (k) and the mRNA concentration (85, 88 and 72% of genes were in phases I and II for the *rph-1* control strain and the *rph-1* Δ*rnr* and *rph-1* Δ*pnp* double mutants*,* respectively) (Table 2). This result shows that in a strain mRNA stability and concentration are generally tightly linked in vivo. However in the *rph-1* Δ*pnp* double mutant*,* a more than 2-fold higher number of genes with uncorrelated degradation rate constant and concentration was observed (1144 genes in the *rph-1* Δ*pnp* double mutant were in the phase III against about 400 genes in the two other strains) (Table 2). Most of the genes in phase III in the *rph-1* Δ*pnp* double mutant (779) belonged to phases I or II in the *rph-1* control strain. This was not the case in the *rph-1* Δ*rnr* double mutant since nearly all the genes found in phase III were also found in this phase in the *rph-1* control.

**Table 2:** Phase by phase analysis of the correlation between the degradation rate constant k and the mRNA concentration for the *rph-1* control strain and the *rph-1* Δ*rnr* and *rph-1* Δ*pnp* double mutants. Number of genes, correlation coefficients and the associated *p*-value are given in the three zones defined in Fig. 3 for the *rph-1* control strain and the *rph-1* Δ*rnr* and *rph-1* Δ*pnp* double mutants

	Data	PHASE I	PHASE II	PHASE III
*rph-1* control	Number of genes	1889	834	476
	Correlation coefficient	−0.80	−0.60	0.27
	*p*-value	4.14E-108	1.07E-16	9.87E-09
*rph-1* Δ*rnr* double mutant	Number of genes	1957	750	369
	Correlation coefficient	−0.80	−0.69	0.04
	*p*-value	9.66E-107	1.88E-23	4.23E-01
*rph-1* Δ*pnp* double mutant	Number of genes	1150	1747	1144
	Correlation coefficient	−0.83	−0.70	0.28
	*p*-value	8.75E-121	1.03E-23	3.29E-09

mRNA concentration was thus negatively correlated to stability for a large majority of mRNAs (phase I and II) even in the *rph-1* Δ*pnp* double mutant which displayed an unexpected swing in mRNA concentrations and stabilities when compared to the *rph-1* control strain. However, the number of mRNAs in phase III with unrelated stability and concentration values increased significantly in the *rph-1* Δ*pnp* double mutant*.* PNPase mutation thus affected significantly the relationship between mRNA concentration and stability unlike the RNase R mutation. This suggests that PNPase is a key enzyme of the RNA degradation machinery and the implication on the regulation of the in vivo kinetics of mRNA degradation will be presented more in details in the discussion section.

## Discussion

RNase R and PNPase are two of the main exoribonucleases that have important roles in the degradation of RNAs in stationary phase. In this study we used the MG1655 background. In this genetic background, the *rph-1* allele results in a truncated functionally inactive RNase PH, a 3′-5′ exoribonuclease primarily involved in tRNA maturation and rRNA degradation [43]. In this work we were able to determine the genome-wide mRNA stabilities for *E. coli* MG1655 (the *rph-1* control strain) cells and for the *rnr* and *pnp* deletion mutants in stationary phase. This is the first report where the genome-wide mRNA half-lives were determined to compare how exoribonucleases affect the stability of all mRNAs in *E. coli* in stationary phase.

Our results showed that the deletion of RNase R did not affect much the overall stability of mRNAs, when compared to the *rph-1* control strain. A wide response affecting most of the mRNA stabilities was however observed in the PNPase mutant. This difference in the two exoribonuclease mutant strains was further supported by our transcriptomic results. They showed that in the RNase R mutant there were only a few differentially expressed transcripts when compared to the *rph-1* control strain, while for the PNPase mutant more than two thousand transcripts were differentially expressed. PNPase and RNase R are both known to be mainly expressed in stationary phase [12, 22, 23]. Single mutants are viable but the double mutant RNase R/PNPase is not viable [16]. From this result, one would expect that in the absence of PNPase, RNase R would compensate and vice versa. Our results argue in favour of the compensation of RNase R loss by PNPase exonuclease activity whereas the reverse does not seem to happen. Unlike RNase R, PNPase is a key enzyme of the *E. coli* degradosome, a multiprotein complex that is able to degrade RNAs very fast due to the cooperation of the different enzymes [44]. There is also the important role of PNPase in the metabolism of sRNAs [22, 28, 45]. So far, RNase R does not appear to have any significant role in sRNA decay [46]. Therefore, PNPase has several roles in the cell that cannot be fully compensated by RNase R and it could provide one possible explanation as to why the deletion of this enzyme has a greater effect on mRNA concentration and half-life in stationary phase, than the deletion of RNase R.

It was expected that in the absence of these ribonucleases the mRNAs would be stabilized. The reality was far different. Indeed only in the PNPase mutant, about two hundred mRNAs were significantly stabilized (more particularly mRNAs from genes involved in the central carbon metabolism). The few mRNAs stabilized in the PNPase mutant attest the degradation activity of PNPase. On the other hand, the general destabilization of bulk mRNAs in the PNPase mutant suggests that PNPase would have also a role in mRNA protection. A parallel could be drawn between this dual role in degradation and protection and the two known antagonistic PNPase’s functions of exoribonuclease and polymerase [7]. An indirect mechanism due to the impact of PNPase over several key RNAs in the cell can be involved in this apparent mRNA protection. The variations of the level of ncRNAs and notably CsrB in absence of PNPase support such an indirect regulation of mRNA stabilities. PNPase is negatively controlled by CsrA through its binding to the *pnp* mRNA, preventing its translation [47]. In reverse, the destabilisation of CsrB by PNPase observed in this work suggests the positive control of CsrA by PNPase. Such opposite regulations create a feedback regulation loop that is likely to coordinate Csr system and PNPase in vivo to tightly control mRNA stabilities. A second mechanism involving direct binding of PNPase on mRNAs can also be assumed to contribute to this apparent protection of mRNAs by PNPase. We have first observed that mRNAs previously demonstrated to physically interact with PNPase [28] are mainly destabilised in the *rph-1* Δ*pnp* double mutant (Table 3). Secondly, RNase II protects mRNA in *E. coli* by removing poly(A) tails and diminishing mRNA accessibility of other exonucleases [48]. Without PNPase, the corresponding mRNA might thus be more prone to degradation by other ribonucleases, including RNase R. This hypothesis is further supported by the presence of *ompA*, a well-studied RNase R target [12] in the list of genes supposedly protected by PNPase. Additional genes protected by PNPase, namely *fliA, thiL, purN* and *ihfA* were identified as RNase R targets in *Pseudomonas putida* [30].

**Table 3:** Distribution of PNPase mRNA targets identified by co-immunoprecipitation (corresponding to the 278 mRNAs listed in Table S3 of reference [28]) in the different classes of mRNAs categorized by their changes in half-life in the *rph-1* Δ*pnp* double mutant vs the *rph-1* control strain (*p*-value < 0.05)

	Bandyra’s target (number)	Bandyra’s target (%)
significantly DESTABILIZED in the *rph-1* Δ*pnp* double mutant	178	65%
significantly STABILIZED in the *rph-1* Δ*pnp* double mutant	8	3%
non significantly DESTABILIZED in the *rph-1* Δ*pnp* double mutant	59	22%
non significantly STABILIZED in the *rph-1* Δ*pnp* double mutant	29	11%
Total	274	100%

The transcriptomic data for PNPase mutant showed that most transcript concentrations were up-regulated compared to the *rph-1* control strain. Our results show that these increases in concentration were probably not due to RNA stabilization since many RNAs were destabilized in the PNPase mutant. To further decipher the roles of PNPase and RNase R, we have analysed the correlation between the genome-wide mRNA concentrations and half-lives within each of the three strains. At low and intermediate mRNA concentrations, a negative relationship between mRNA concentration and degradation rate constant existed which is consistent with other omics works performed in the MG1655 strain of *E. coli* and wild-type strains of other micro-organisms [2, 36, 49, 50]. Recently it was shown that the stability of an mRNA can be influenced by its concentration and therefore substrate-enzyme interactions can govern this negative relationship [42]. The correlation represents in fact the strong dependency of in vivo degradation activity on the mRNA concentration. When the mRNA concentration is low, the degradation machinery is limited by the substrate availability. However, at high mRNA concentration the degradation rate constant and the mRNA concentration were not correlated anymore. The in vivo mRNA degradation became nearly independent of the mRNA concentration revealing an unexpected phenomenon of enzymatic saturation at high mRNA concentration. It is thus possible to speculate that when the mRNA concentration is low, the in vivo degradation activity is limited by the substrate availability while when the mRNA concentration is high the in vivo degradation activity is more prone to be limited by the enzyme (RNase) activity/quantity. Therefore, in the *rph-1* control strain, the mRNA degradation appeared to be mainly limited in vivo by the mRNA concentration and more rarely by the in vivo degradation activity. A similar behavior was found for the RNase R mutant. In the contrary, in the PNPase mutant*,* mRNA degradation was more frequently limited by the in vivo degradation activity. Deletion of the PNPase activity was thus demonstrated to shape the RNA degradation towards a substrate saturation of the degradation machinery. This could be a direct consequence of the *pnp* deletion on the in vivo degradation activity or due to indirect changes in mRNA concentration provoked by the mutation. PNPase was thus evidenced as a central player of the degradation machinery.

## Conclusions

The combined analysis (between two strains or within the same strain) of the genome-wide mRNA concentrations and half-lives has provided substantial knowledge on the impact of PNPase activity on the mRNA metabolism. The understanding of the mRNA concentration and degradation relationship allows a wider comprehension of the in vivo activity of RNase pointing out the crucial role of PNPase. In the future, such an approach connecting mRNA degradation, mRNA concentration and the exoribonuclease activity will allow the role and the complementarity of several other RNases to be elucidated.

## Methods

### Strains and growth conditions

The double *E. coli* mutant strains with *rnr* or *pnp* deletion (*rph-1* ∆*rnr* and *rph-1* ∆*pnp,* respectively) in the MG1655 background (F^−^ λ^−^*rph-1*) were respectively obtained by phage transduction from donor strains in the MG1693 background (F^−^ λ^−^*rph-1*) [12, 32, 51]. P1-mediated transductions were performed as previously described [52]. 50 μL of an overnight culture of the donor strains were used to inoculate 5 mL of LB with 0.4% glucose and 10 mM CaCl_2_. After 30 min of growth at 37 °C, phage P1 lysate (100 μL) was added. After 2 h incubation at 37 °C, cell lysis was performed by chloroform (100 μl) addition. Cells were centrifuged at 13500 rpm for 10 min, the supernatant (transducing lysate) transferred to a new tube with 30 μl chloroform and kept at 4 °C. An overnight culture of the recipient strain was centrifuged and the pellet resuspended in 2.5 ml LB with 10 mM MgSO_4_ and 5 mM CaCl_2_. Cells (100 μl) were incubated with 10 to 200 μl of transducing lysate during 30 min at 30 °C. 300 μl of sodium citrate 1 M were added and the cells were incubated another hour at 30 °C. Infected cells were centrifuged and resuspended in 100 μL of the supernatant. Transductants were selected by plating on LB agar plates containing kanamycin (50 μg·ml^− 1^) for the *rph-1* ∆*rnr* strain and streptomycin (50 μg·ml^− 1^) for the *rph-1* ∆*pnp* strain. The *rph-1* control strain and the *rph-1* ∆*rnr* and *rph-1* ∆*pnp* double mutants were grown in baffled flasks in LB medium at 37 °C and 180 rpm. Initial pH was set to 7. All cultures were inoculated at a low OD of 0.1 to maintain cells in exponential phase of growth for many generations prior to entry into stationary phase to dilute out any RNAs and proteins provided by the inoculum. Inoculation was performed from overnight pre-cultures performed in similar conditions. Biomass was estimated from absorbance at 600 nm. Each culture was repeated three times to provide independent biological replicates.

### RNA extraction protocol

Three hours after the stationary phase of growth was reached, samples were collected for transcriptomic analysis; this time point was also the reference time point (T0) for the half-life determination procedure. Subsequently, rifampicin (500 μg.mL^− 1^) was added to inhibit the initiation of transcription, and cells were harvested at three different time points after this addition. Cultures were performed in triplicate. Samples were taken at either at 0′, 2′, 8′ and 15′, or 0′, 5′, 10′ and 18′ or 0′, 3′, 12′ and 30′ min after rifampicin addition. Samples were immediately frozen in liquid nitrogen upon collection. After thawing and centrifugation steps, total RNA was extracted with TRIZOL® Reagent (Ambion) according to the manufacturer’s instructions. DNA contamination was eliminated with Turbo DNase kit (Ambion). Total RNA concentration and integrity were measured using a Nanodrop® spectrophotometer and Agilent BioAnalyzer, respectively. Total RNA extraction profiles were checked to be similar for all the three tested strains.

### Microarray procedures

A double-stranded cDNA synthesis kit (InvitroGen) was used to produce cDNA from 2 μg aliquots of total RNA. Aliquots of 1 μg of cDNA were labeled using the one color DNA labeling kit and 2 μg of labeled cDNA were hybridized onto *E. coli* K-12 gene expression arrays (Nimblegen, Roche) for 17 h at 42 °C according to the manufacturers’ instructions. Arrays were washed and then scanned with a MS200 Microarray Scanner (Nimblegen, Roche). The images were analyzed with DEVA 1.2.1 software. Only raw data were used for further analyses. All array procedures were performed by the GeT-Biopuces platform (http://get.genotoul.fr).

### Transcriptome and genome-wide mRNA half-life measurements

For transcriptomic analysis, raw probe intensities were processed and analyzed with the R computing environment using the affy [53] and limma [54] packages of Bioconductor. Raw data were submitted to a RMA-based background correction [55]. After background correction, intra-replicate quantile normalization was performed for each strain. A set of probes in the background for which the ranks were roughly invariant across all nine arrays was selected. The median value of the invariant probe set intensities in each condition was used as a scaling factor for normalization between the three strains. After normalization, the intensity of a transcript was calculated by a RMA-summarization procedure [55] within each condition. Intensity values were multiplied by the total RNA extraction yield (in μg total RNA per mg of dry cell weight) to provide the mRNA concentration value in arbitrary units per mg of dry cell weight. RNA extraction yields were 15.6 ± 3.0, 17.2 ± 2.3 and 35.9 ± 7.3 μg RNA per mg of dry cell weight for growth of the *rph-1* control strain and the *rph-1* ∆*rnr* and *rph-1* ∆*pnp* double mutants, respectively. The multiplication step by the total RNA extraction yield allowed to take into account differences in total RNA content (rRNA, tRNA and mRNA) per cell weight between the strains. Differences in mRNA concentration were evaluated with a modified t-test in conjunction with an empirical Bayes method [54]. The *p*-values were adjusted for multiple testing by the “BH” False Discovery Rate (FDR) method [56]. A p-value threshold of 1% and a fold change higher than 2 or lower than 0.5 were used for significance of differences in mRNA concentration.

For mRNA half-life determinations, twelve arrays (three reference T0 samples, and nine time points after addition of rifampicin) were used. Only the normalization between arrays according to the invariant probeset intensities was performed. In each array, transcript-specific intensity was computed as the median value of the 16 targeting probe intensities. The linear regression coefficient, k, of ln(mRNA) versus time (12 points) and its associated coefficient of variation (standard error of slope/estimation of slope) were calculated for each mRNA species. The determination of k was considered as reliable only if the associated coefficient of variation was below 30%. The linear regression coefficient k corresponding to the degradation rate constant was inversely proportional to the mRNA half-life t_1/2_, $k=\frac{\ln 2}{{\mathrm{t}}_{1/2}}$. The statistical significance of differences in half-life was evaluated using the probability value of interaction between time and growth rate in a global model of linear regression. A statistical threshold of 10% was used for adjusted *p*-values by the “BH” FDR method [56].

For functional analyses, GO and KEGG enrichment analyses were performed on the differentially expressed genes as well as on the genes with differential stabilities. Enrichment significance was set with a cut-off of 5% for the associated *p*-value.

### ncRNAs were quantified by RNA-Seq technology in the *rph-1* Δ*pnp* double mutant and the *rph-1* control strain

RNA samples (20 μg) were sent to Vertis Biotechnologie AG, Germany, for library preparation and sequencing of libraries using an Illumina HiSeq platform (single end, 50-bp read length). For the library preparation Vertis Biotechnologie AG depleted the ribosomal RNA molecules from the total RNA preparations using the MICROBExpress Bacterial mRNA Enrichment Kit (Ambion). The rRNA depleted RNAs were then fragmented with RNase III and the 5’PPP structures were removed using RNA 5’ Polyphosphatase (Epicentre). Afterwards, the RNA fragments were poly(A)-tailed using poly(A) polymerase and a RNA adapter was ligated to the 5′-phosphate of the RNA fragments. First-strand cDNA synthesis was performed using an oligo(dT)-adapter primer and M-MLV reverse transcriptase. The resulting cDNA was PCR-amplified to about 30 ng/μl using a high fidelity DNA polymerase and sequenced. The reads were mapped against *E. coli* genome (NC_000913 downloaded from NCBI genome database). The ncRNAs expression was quantified using Artemis [57] and the Log2 Fold change was calculated for each of the ncRNAs.

### Confirmation of mRNA half-life data by northern blot

Three genes *osmB*, *ompA* and *nlpl* were selected for additional calculation of their associated mRNA half-life using Northern blot method. Northern blots were performed using the total RNA samples extracted for microarrays. Briefly, 10–30 μg of total RNA were fractionated under denaturing conditions in 1.2% agarose formaldehyde gel in MOPS buffer. RNAs were transferred onto Hybond-N+ membrane (GE Healthcare) and cross-linked by UV irradiation using a UVC 500 apparatus (Amersham Biosciences). Membranes were hybridized overnight with radiolabeled specific probes in PerfectHyb Plus Hybridization Buffer (Sigma Aldrich) at 68 °C. Specific probes (primers are given in Additional file 4: Figure S4) were obtained by in vitro transcription reactions with PCR DNA templates carrying a T7 promoter sequence through the use of 32P-UTP and T7 RNA polymerase (Promega). Radiolabeled probes were purified on G25 Microspin columns (GE Healthcare).

### Availability of supportive data

The data discussed in this publication have been deposited in NCBI’s Gene Expression Omnibus [58] and are accessible through GEO SuperSeries accession number GSE116652 and the GEO Series GSE60107 for microarrays and RNA-Seq data, respectively. Other supportive data are included as additional files.

## Additional files

Additional file 1: **Figure S1.** Growth curves. The semilog plot shows the mean value and the standard deviation of three independent cultures. The sampling point in stationary phase is shown by an arrow. (A). The *rph-1* control, (B) the *rph-1* Δ*rnr* double mutant and (C) the *rph-1* Δ*pnp* double mutant. (PPTX 91 kb)

Additional file 2: **Figure S2.** mRNA half-life measurement with classical linear model fitted on decay of log concentration of mRNA over the time (expressed in minutes). Example of 50 random mRNAs selected among the 2856 available for all the three strains (Blue = the *rph-1* control, Green = the *rph-1* Δ*pnp* double mutant and Red = the *rph-1* Δ*rnr* double mutant). (PPTX 91 kb) (PPTX 430 kb)

Additional file 3: **Figure S3.** Correlation of half-lives with and without delays in the three strains. The coefficient of correlation is 1 and the slope of the linear model 0.98. (JPG 553 kb)

Additional file 4: **Figure S4.** Confirmation of mRNA half-life data by Northern blot experiments. Three mRNAs, *ompA*, *osmB* and *nlpI* were selected in the three strains for mRNA half-life measurements by Northern blot experiments. The list of primers used is given in the table and the T7 promoter sequences in the oligonucleotides are in bold. (PPTX 58 kb)

Additional file 5: **Table S1.** Selection of transcriptional regulators with a modified stability in the *rph-1* Δ*pnp* double mutant. Fold-change (FC) of half-lives in the *rph-1* Δ*pnp* double mutant compared to the *rph-1* control strain is given with the associated *p*-value. The 6 stabilized mRNAs in the *rph-1* Δ*pnp* double mutant are in red whereas the highest destabilized mRNAs defined with FC < 0.3 are in green. (DOCX 50 kb)

Additional file 6: **Table S2.** Total RNA yield and mRNA concentration in the 3 strains. AU: arbitrary unit, DCW: dry cell weight. (DOCX 39 kb)
