# Supplementary figures and images for: PNPase is involved in the coordination of mRNA degradation and expression in stationary phase cells of Escherichia coli
Source: BMC Genomics. 2018 Nov 29;19:848. doi: 10.1186/s12864-018-5259-8 (PMC6264599; doi:10.1186/s12864-018-5259-8)

## Slide 1
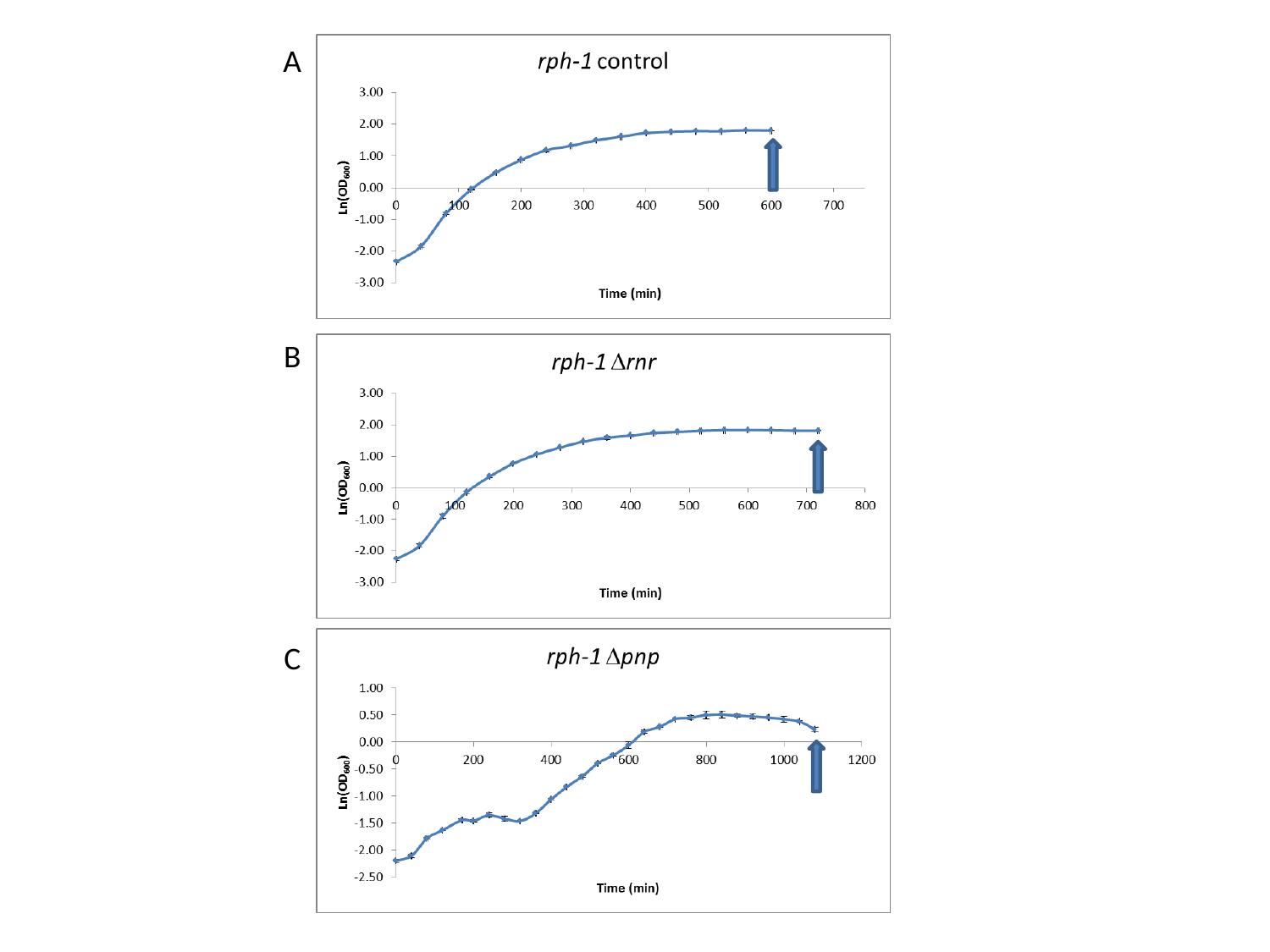

A
B
C

Supplement: Supplementary file 1 — Figure S1. Growth curves. The semilog plot shows the mean value and the standard deviation of three independent cultures. The sampling point in stationary phase is shown by an arrow. (A). The rph-1 control, (B) the rph-1 Δrnr double mutant and (C) the rph-1 Δpnp double mutant. (PPTX 91 kb) [file 12864_2018_5259_MOESM1_ESM.pptx]

## Slide 1
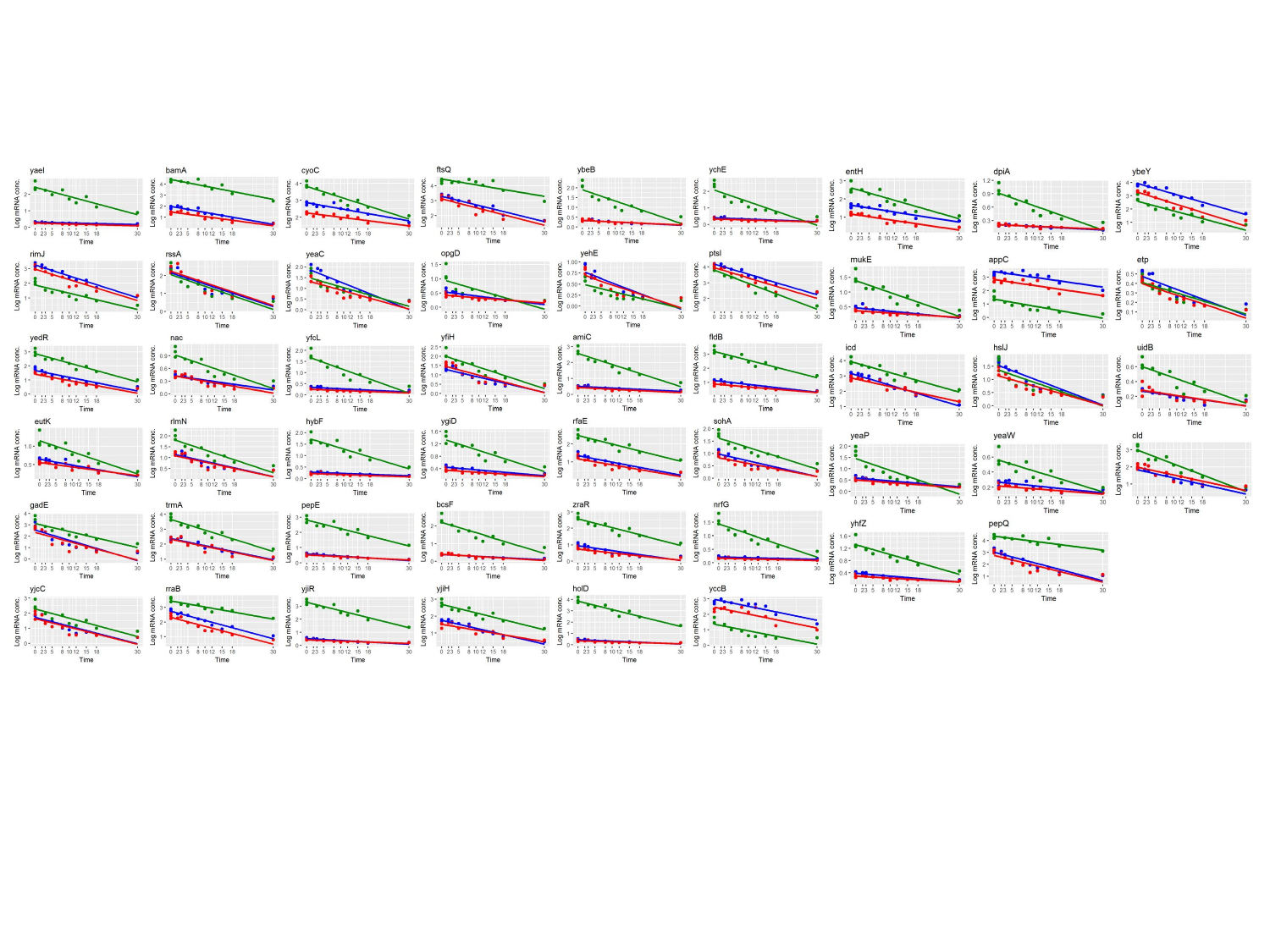

Supplement: Supplementary file 2 — Figure S2. mRNA half-life measurement with classical linear model fitted on decay of log concentration of mRNA over the time (expressed in minutes). Example of 50 random mRNAs selected among the 2856 available for all the three strains (Blue = the rph-1 control, Green = the rph-1 Δpnp double mutant and Red = the rph-1 Δrnr double mutant). (PPTX 91 kb) (PPTX 430 kb) [file 12864_2018_5259_MOESM2_ESM.pptx]

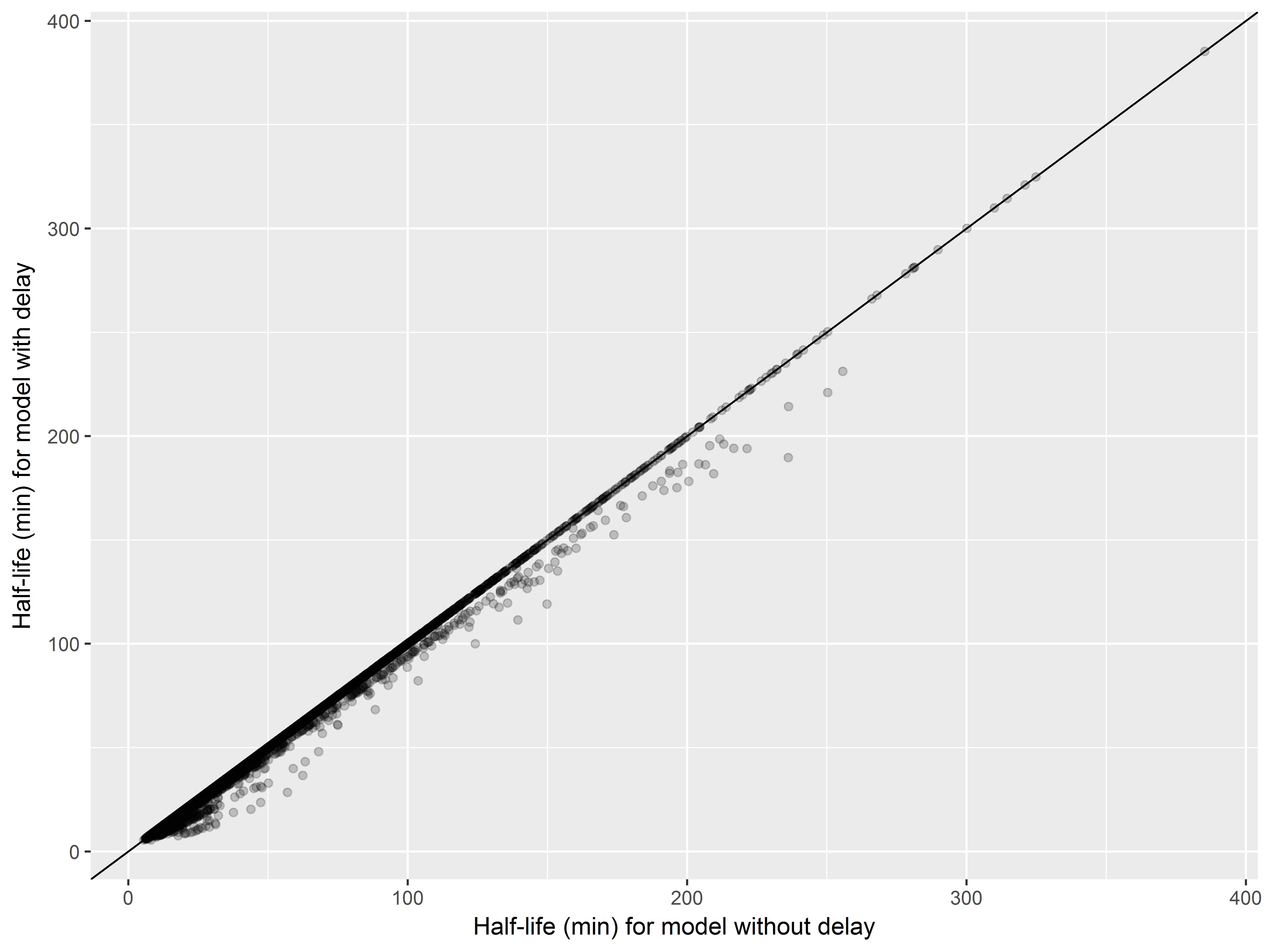

Supplement: Supplementary file 3 — Figure S3. Correlation of half-lives with and without delays in the three strains. The coefficient of correlation is 1 and the slope of the linear model 0.98. (JPG 553 kb) [file 12864_2018_5259_MOESM3_ESM.jpg]

## Slide 1
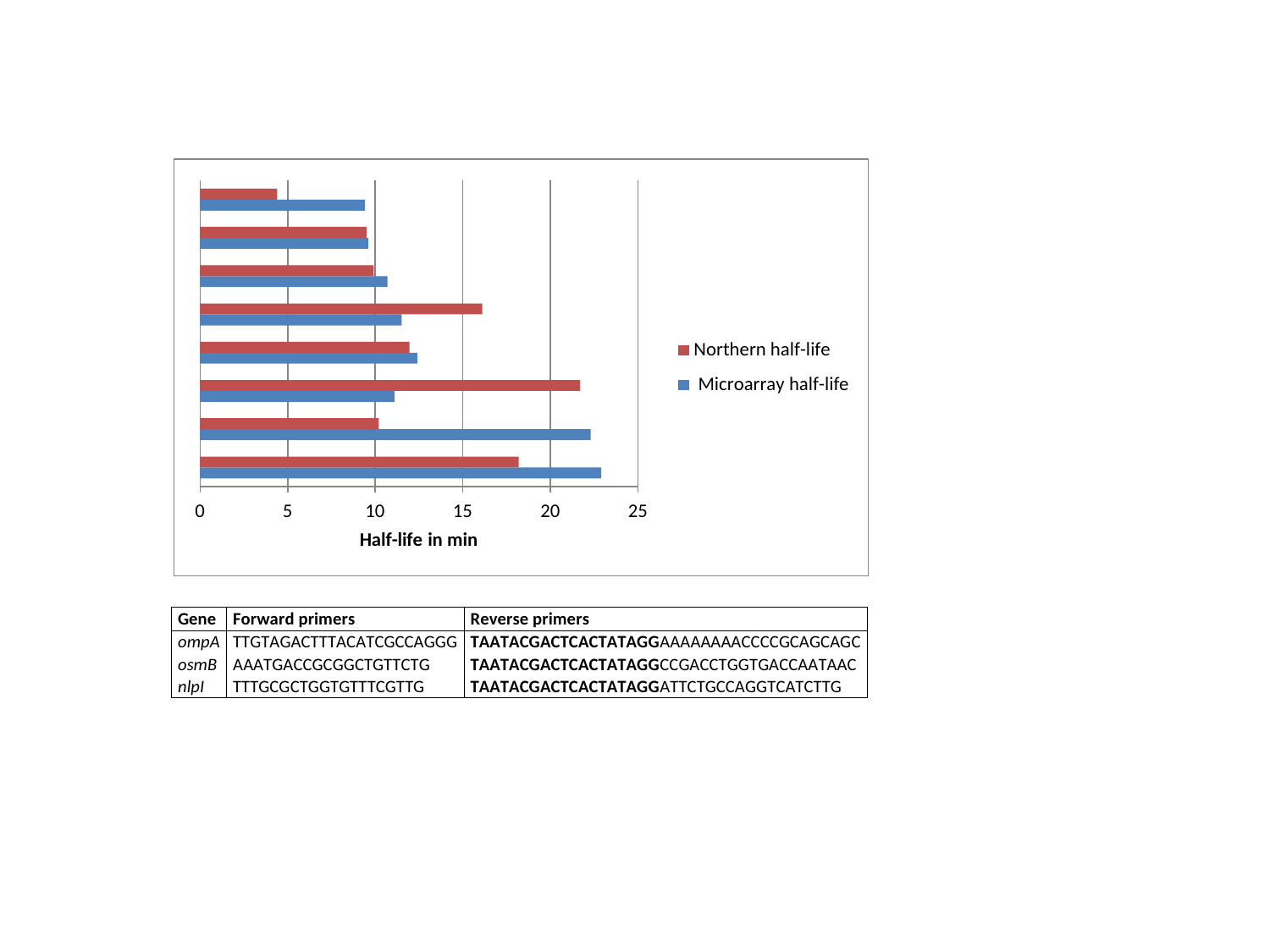

Supplement: Supplementary file 4 — Figure S4. Confirmation of mRNA half-life data by Northern blot experiments. Three mRNAs, ompA, osmB and nlpI were selected in the three strains for mRNA half-life measurements by Northern blot experiments. The list of primers used is given in the table and the T7 promoter sequences in the oligonucleotides are in bold. (PPTX 58 kb) [file 12864_2018_5259_MOESM4_ESM.pptx]
