# Supplementary material for: PNPase is involved in the coordination of mRNA degradation and expression in stationary phase cells of Escherichia coli
Source: BMC Genomics. 2018 Nov 29;19:848. doi: 10.1186/s12864-018-5259-8 (PMC6264599; doi:10.1186/s12864-018-5259-8)
Supplement: Supplementary file 5 — Table S1. Selection of transcriptional regulators with a modified stability in the rph-1 Δpnp double mutant. Fold-change (FC) of half-lives in the rph-1 Δpnp double mutant compared to the rph-1 control strain is given with the associated p-value. The 6 stabilized mRNAs in the rph-1 Δpnp double mutant are in red whereas the highest destabilized mRNAs defined with FC < 0.3 are in green. (DOCX 50 kb) [file 12864_2018_5259_MOESM5_ESM.docx]

**Table S1.docx:** Selection of transcriptional regulators with a modified stability in the *rph-1* Δ*pnp* double mutant. Fold-change (FC) of half-lives in the *rph-1* Δ*pnp* double mutant compared to the *rph-1* control strain is given with the associated p-value. The 6 stabilized mRNAs in the *rph-1* Δ*pnp* double mutant are in red whereas the highest destabilized mRNAs defined with FC< 0.3 are in green.
