# Supplementary material for: PNPase is involved in the coordination of mRNA degradation and expression in stationary phase cells of Escherichia coli
Source: BMC Genomics. 2018 Nov 29;19:848. doi: 10.1186/s12864-018-5259-8 (PMC6264599; doi:10.1186/s12864-018-5259-8)
Supplement: Supplementary file 6 — Table S2. Total RNA yield and mRNA concentration in the 3 strains. AU: arbitrary unit, DCW: dry cell weight. (DOCX 39 kb) [file 12864_2018_5259_MOESM6_ESM.docx]

**Table S2.docx**: Total RNA yield and mRNA concentration in the 3 strains. AU: arbitrary unit, DCW: dry cell weight.

| Strain | Total RNA yield in µg /mg DCW | | | mRNA concentration in AU/mg DCW | mRNA concentration in % of WT |
| --- | --- | --- | --- | --- | --- |
| *rph-1* control | 15.6 | ± | 3.0 | 4.18E+08 | 100 |
| *rph-1* Δ*rnr* | 17.2 | ± | 2.3 | 3.53E+08 | 84 |
| *rph-1* Δ*pnp* | 35.9 | ± | 7.3 | 2.01E+09 | 480 |
